# Can pre-diagnostic serum levels of sodium and potassium predict prostate cancer survival?

**DOI:** 10.1186/s12885-018-5098-7

**Published:** 2018-11-26

**Authors:** Arunangshu Ghoshal, Hans Garmo, Niklas Hammar, Ingmar Jungner, Håkan Malmström, Göran Walldius, Mieke Van Hemelrijck

**Affiliations:** 1 Translational Oncology & Urology Research, Kings’s College London, School of Cancer and Pharmaceutical Sciences, 3rd Floor, Bermondsey Wing, Guy’s Hospital, London, SE1 9RT UK; 2 Regional Cancer Centre, Uppsala University, Box 256 751 05 Uppsala, Sweden; 3 Unit of Epidemiology, Institute of Environmental Medicine, Karolinska Institutet, SE-171 77 Stockholm, Sweden; 4 AstraZeneca R&D, 431 50 Mölndal, Sweden; 5 Department of Medicine, Clinical Epidemiological Unit, Karolinska Institutet and CALAB Research, SE-171 77 Stockholm, Sweden; 6 Biostatistics, Research & Development, Swedish Orphan Biovitrum AB, SE-112 76 Stockholm, Sweden; 7 Unit of Cardiovascular Epidemiology, Institute of Environmental Medicine, Karolinska Institutet, SE-171 77 Stockholm, Sweden; 8 Department of Palliative Medicine, Tata Memorial Hospital, Mumbai, 40012 India

**Keywords:** AMORIS cohort, Prospective study, Prostate cancer, Serum electrolytes, Survival analysis

## Abstract

There is evidence that derangement in serum electrolytes like sodium and potassium is associated with increased morbidity and mortality among hospitalized critically ill patients, but their role in the context of cancer survival remains poorly understood. We sought to investigate the association of pre-diagnostic serum sodium and potassium with risk of overall, cancer-specific, and cardiovascular (CV) death among 11,492 men diagnosed with prostate cancer (PCa) from the Swedish AMORIS study. Multivariable Cox proportional hazards regression was used to assess the risk of death by clinical categories of pre-diagnostic serum sodium and potassium. During a mean follow-up of 5.7 years, 1649 men died of PCa. Serum levels of sodium were not indicative of PCa-specific or CV death. A weak positive association was found between pre-diagnostic higher serum potassium (> 5 mEq/L) and overall death [HR: 1.26 (95% CI: 1.01–1.59)] as compared to low/normal levels of clinical cut-offs. The current study did not find strong evidence for a role of electrolytes in PCa mortality. To further disentangle the potential role of electrolytes in cancer development, future studies should use repeated measurement of serum electrolytes.

This research project was reviewed and approved by the Stockholm Ethical Committee (Dnr 2010/1:7).

## Introduction

Fluid and electrolyte imbalances are thought to be associated with increased morbidity and mortality among hospitalized critically ill (cancer) patients [1, 2]. They have also been extensively studied in relation to cardiovascular-specific death [3]. Even though serum electrolytes like sodium (Na) and potassium (K) are routinely measured in clinical practice, most studies to date are based on hospital data with these measurements taken at time of diagnosis or treatment initiation [4]. However, the effects of electrolyte imbalances may influence health outcomes over a much longer time. In a study by Verma A, et al. [5] using the National Health and Nutrition Examination survey (NHANES) data (1999–2010), a higher serum anion gap was associated with increase in cancer mortality. A recent systematic review also highlighted the need to understand the potential role of these electrolytes in the context of carcinogenesis [6].

Prostate cancer (PCa) is the most common non-cutaneous cancer in men. Although PCa can be a slow-growing cancer, it is the second most common cause of cancer death in males globally [7]. The most important and established indicators of prognosis for PCa include Gleason grade, extent of tumour volume, and presence of capsular penetration or margin positivity at time of prostatectomy [8]. To our knowledge, there are no studies yet evaluating the effect of pre-diagnostic electrolyte imbalances on PCa mortality.

Hence, we sought to investigate the association between pre-diagnostic serum Na and K and overall, cancer-specific, and cardiovascular death among 11,492 men with PCa in a prospective cohort study with up to 25 years of follow-up.

## Methods

### Study population and data collection

The Swedish Apolipoprotein-related MOrtality RISk database (AMORIS) includes blood samples from 812,073 Swedish men and women, ranging in age from < 20 to 80 + years undergoing occupational health screening or primary care. The cohort is based on a linkage between data from laboratory examinations performed in the Central Automation Laboratory (CALAB) in Sweden and information recorded in Swedish National Registers using a 10-digit personal identifier number, which is unique to every Swedish resident. Individuals recruited were primarily from the greater Stockholm area, who were either healthy and having laboratory testing as part of a general checkup or outpatients referred for laboratory testing. None of the participants were inpatients at the time of sampling. In the AMORIS cohort, the CALAB database was linked to several Swedish national registries such as the Swedish National Cancer Register, the Hospital Discharge Register, the Cause of Death Register, the consecutive Swedish Censuses during 1970–1990, and the National Register of Emigration using the Swedish 10-digit personal identity number [9, 10]. Detailed description of the AMORIS cohort can be found elsewhere [11].

For this study, we specifically focused on the linkage between the AMORIS database and the National Prostate Cancer Register (NPCR), which has been nationwide since 1998 [12]. NPCR was developed to provide data for quality assurance and includes 98% of all newly diagnosed PCa cases registered in the Swedish National Cancer Register [12] to which reporting is mandated. From the NPCR, we extracted information on date of diagnosis, age at diagnosis, TNM stage [13], Gleason score, serum concentration of PSA at time of diagnosis, and primary treatment given or planned up to 6 months after date of diagnosis. Information on educational level was retrieved from the Population and Housing Census for 1970–1990. Using information from the National Patient Register, we calculated the Charlson Comorbidity Index which includes 19 diseases, with each disease category assigned a weight. The sum of an individual’s weights was used to create a score, resulting in four comorbidity levels ranging from no comorbidity to severe comorbidity (0, 1, 2, and ≥ 3) [14]. AMORIS is a large prospective cohort with information on serum biomarkers, cancer diagnosis, co-morbidities, vital status, socioeconomic status, and emigration. This study complied with the Declaration of Helsinki and was approved by the Ethics Review Board of the Karolinska Institute [11].

We included all men aged 20+ years diagnosed with no previous diagnosis of any cancer, and measurements of serum Na and K available from the same health examination on average 13 years [interval time (range: 0.01–25.9 years)] prior to the diagnosis of PCa (*n* = 11,492). Na and K were assessed based on their standard clinical cut-offs in serum (Na: 136–145 mEq/L, K: 3.5–5 mEq/L) [15]. Follow-up time was defined as time from PCa diagnosis until date of cancer death, date of death, emigration, or end of study (31st December 2011), whichever came first. The primary outcome investigated in this study was death from prostate cancer (International Classification of Diseases, Revision 7 (1955) code 177), as registered in the National Cause of Death Register [16].

We estimated risk of death with multivariate Cox proportional hazards regression in relation to serum levels of Na and K, whilst adjusting for interval time (i.e. time between measurement and diagnosis), and the following information from AMORIS: age, educational level (low, intermediate, high), Charlson Comorbidity Index, PCa severity (defined by PCa risk categories^1^ and clinicopathological characteristics (i.e., PSA, Gleason score, and TNM staging) in the updated AMORIS cohort [17]): low, intermediate, and high), and serum creatinine (μmol/L) (continuous). CCI was calculated using information from the National Patient Register and consists of 18 groups of diseases with a specific weight assigned to each disease category, summed to obtain an overall score, resulting in four comorbidity levels (0, 1, 2, and ≥ 3) [18]. We also created a combined electrolyte score by adding the clinical cut-off variables for Na and K. The score ranged from 0 to 4 (0,1: low levels of Na and/or K, 2: normal levels of Na and/or K, 3,4: high levels of Na and/or K). Additionally, we performed stratified analyses by quartiles of age at baseline (36, 64, 69, and 75 years) and interval time (0.01, 9, 14, and 18 years), and sensitivity analyses excluding PCa death < 3 years and < 5 years after PCa diagnosis.

All analyses were conducted with Statistical Analysis Systems (SAS) release 9.4 [19]. The assumption of proportionality was checked using the methods of Lin, Wei, and Ying [20]. This study complied with the Declaration of Helsinki and was approved by the Ethics board of the Karolinska Institute.

## Results

During a mean follow-up time of 5.7 years [range: (0.01–24.5 years)], 1649 men died of PCa and 485 of cardiovascular diseases out of 3995 deaths. Baseline characteristics of the study participants are shown in Table 1. Most subjects had value of Na and K in the middle categories of Na and K.

**Table 1:** Descriptive statistics of the study population (men diagnosed with Prostate Cancer in AMORIS) (*n* = 11,492)

	Sodium (mEq/L) Mean = 140.71 (SD = 2.92)			Potassium (mEq/L) Mean = 4.26 (SD = 0.33)		
	< 136 (*n* = 255)	136–145 (*n* = 10,584)	> 145 (*n* = 653)	< 3.5 (*n* = 47)	3.5–5 (n = 11,268)	> 5 (*n* = 177)
Mean age (yrs.) (SD)	69.7 (8.3)	69.5 (8.1)	69.4 (8.0)	70.4 (8.5)	69.5 (8.1)	70.7 (8.6)
Socio-economic status (%)						
White collar	152 (59.61)	7145 (67.51)	455 (69.68)	29 (61.7)	7609 (67.53)	114 (64.41)
Blue collar	85 (33.33)	2903 (27.43)	169 (25.88)	17 (36.17)	3095 (27.47)	45 (25.42)
Unemployed/missing	18 (7.06)	536 (5.06)	29 (4.44)	1 (2.13)	564 (5.01)	18 (10.17)
Education (%)						
Low	78 (30.59)	2782 (26.28)	178 (27.26)	18 (38.29)	2979 (26.44)	41 (23.16)
Middle	102 (0.4)	4323 (40.84)	257 (39.36)	17 (36.17)	4584 (40.68)	81 (45.76)
High	152 (59.61)	3137 (29.64)	202 (30.93)	11 (23.4)	3351 (29.74)	46 (25.99)
Missing	6 (2.35)	342 (3.23)	16 (2.45)	1 (2.13)	354 (3.14)	9 (5.08)
Charslon Co-morbidity Index (%)						
0	160 (62.74)	6947 (65.64)	435 (66.62)	28 (59.57)	7411 (65.77)	103 (58.19)
1	37 (14.51)	1464 (13.83)	84 (12.86)	10 (21.28)	1544 (13.7)	31 (17.51)
2	33 (12.94)	1325 (12.52)	84 (12.86)	7 (14.89)	1414 (12.55)	21 (11.86)
3+	25 (9.80)	848 (8.01)	50 (7.66)	2 (4.26)	899 (7.98)	22 (12.43)
Body mass index (kg/m^2^) (%)						
< 18.5	–	9 (0.08)	–	–	9 (0.08)	–
18.5–24.99	28 (10.98)	919 (8.68)	62 (9.49)	4 (8.51)	989 (8.78)	16 (9.04)
25–29.99	20 (7.84)	932 (8.80)	55 (8.42)	5 (10.64)	991 (8.79)	11 (6.21)
> = 30	5 (1.96)	195 (1.84)	11 (1.68)	1 (2.13)	208 (1.84)	2 (1.13)
Missing	202 (79.21)	8529 (80.58)	525 (80.39)	37 (78.72)	9071 (80.5)	148 (83.61)
Creatinine (micromol/L)						
Mean (SD)	89.39 (16.47)	89.80 (18.95)	91.66 (12.47)	90.89 (15.29)	89.63 (13.49)	106.98 (103.33)
Missing (%)	1 (0.39)	59 (0.56)	2 (0.31)	0	59 (0.52)	3 (1.69)
Interval time (years)						
Mean (SD)	13.3 (6.6)	13.2 (6.3)	14.3 (5.9)	11.3 (7.2)	13.3 (6.3)	12.1 (6.6)
Cancer severity (%)						
Low risk	49 (19.21)	2337 (22.08)	157 (24.04)	12 (25.53)	2493 (22.12)	38 (21.47)
Intermediate risk	50 (19.61)	2299 (21.72)	139 (21.29)	9 (19.15)	2445 (21.69)	34 (19.21)
High risk	46 (18.04)	1917 (18.11)	109 (16.69)	7 (14.89)	2035 (18.06)	30 (16.95)
Regionally metastatic	14 (5.49)	511 (4.83)	38 (5.82)	–	559 (4.96)	4 (2.26)
Distant metastases	20 (7.84)	814 (7.69)	56 (8.57)	3 (6.38)	870 (7.72)	17 (9.6)
Missing	76 (29.80)	2706 (25.57)	154 (23.58)	16 (34.04)	2866 (25.43)	54 (30.51)

When using the clinical cut-off of serum Na and K as well as a combined electrolyte score, we only observed a weak positive association with all-cause mortality for higher serum levels of K [HR: 1.26 (95% CI: 1.01–1.59)], as compared to low levels according to the medical cut-off (Table 2). No meaningful associations were observed between serum Na levels or combined electrolyte score and PCa-specific mortality, Overall mortality and Cardiovascular mortality. Additional adjustments for creatinine levels, time between measurement and PCa diagnosis, and PCa severity weakened this association: HR 1.11 (95% CI: 0.79–1.55). Stratified analyses by age and interval time and sensitivity analyses excluding rapidly fatal PCa did not alter the above-described associations (results not shown).

**Table 2:** Hazard ratio (HR) for death with 95% confidence intervals (CI) using Cox proportional hazards models

Variables		Prostate Cancer specific mortality			Overall mortality			Cardiovascular mortality		
		Crude	Adjusted ^a^	Adjusted ^b^	Crude	Adjusted ^a^	Adjusted ^b^	Crude	Adjusted ^a^	Adjusted ^b^
		HR (95% CI)	HR (95% CI)	HR (95% CI)	HR (95% CI)	HR (95% CI)	HR (95% CI)	HR (95% CI)	HR (95% CI)	HR (95% CI)
Events		1649		1003	3995		2234	485		302
Sodium (mEq/L)										
Medical cut offs	< 136	1.12 (0.82–1.53)	1.14 (0.84–1.57)	1.15 (0.75–1.75)	1.16 (0.95–1.42)	1.20 (0.99–1.46)	1.14 (0.86–1.51)	1.21 (0.70–2.11)	1.29 (0.75–2.25)	0.82 (0.34–1.98)
	136–145	1.00 (Ref)	1.00 (Ref)	1.00 (Ref)	1.00 (Ref)	1.00 (Ref)	1.00 (Ref)	1.00 (Ref)	1.00 (Ref)	1.00 (Ref)
	> 145	1.08 (0.88–1.32)	1.11 (0.90–1.35)	1.08 (0.84–1.39)	1.00 (0.88–1.14)	1.04 (0.91–1.19)	1.06 (0.89–1.26)	0.75 (0.49–1.17)	0.81 (0.52–1.25)	0.84 (0.49–1.44)
Potassium (mEq/L)										
Medical cut offs	< 3.5	0.65 (0.24–1.72)	0.58 (0.22–1.55)	0.49 (0.12–1.97)	1.19 (0.75–1.90)	1.07 (0.67–1.69)	0.72 (0.34–1.51)	0.72 (0.34–1.51)	1.29 (0.42–4.04)	0.59 (0.08–4.23)
	3.5–5	1.00 (Ref)	1.00 (Ref)	1.00 (Ref)	1.00 (Ref)	1.00 (Ref)	1.00 (Ref)	1.00 (Ref)	1.00 (Ref)	1.00 (Ref)
	> 5	1.21 (0.84–1.75)	1.18 (0.82–1.70)	1.26 (0.76–2.09)	1.33 (1.06–1.67)	1.26 (1.01–1.59)	1.11 (0.79–1.55)	1.11 (0.79–1.55)	1.57 (0.89–2.79)	0.98 (0.39–2.44)
Combined electrolyte scoring (0–4)										
Scoring	Low score (0,1)	1.01 (0.74–1.38)	1.02 (0.75–1.39)	1.04 (0.69–1.57)	1.16 (0.97–1.40)	1.18 (0.98–1.42)	1.06 (0.81–1.39)	1.25 (0.75–2.09)	1.27 (0.76–2.12)	0.65 (0.27–1.57)
	Score 2	1.00 (Ref)	1.00 (Ref)	1.00 (Ref)	1.00 (Ref)	1.00 (Ref)	1.00 (Ref)	1.00 (Ref)	1.00 (Ref)	1.00 (Ref)
	High score (3,4)	1.13 (0.94–1.36)	1.14 (0.95–1.37)	1.13 (0.89–1.42)	1.08 (0.96–1.22)	1.10 (0.98–1.24)	1.06 (0.91–1.25)	0.91 (0.63–1.32)	0.94 (0.65–1.36)	0.84 (0.52–1.37)

^a^adjusted for age, education, Charslon Comorbidity Index

^b^adjusted for age, education, Charslon Comorbidity Index, creatinine (continuous), interval time, disease severity

## Discussion

Pre-diagnostic Na levels were not associated with PCa death. Although higher serum potassium was associated with an increased risk of all-cause mortality, the associations were weak without any clear trend. Data from in-vitro, in-vivo models and ecological studies suggest that serum Na/K represent a potentially modifiable exposure that could affect PCa mortality [21]. Even in clinical studies, electrolyte disorders are commonly encountered in cancer patients, and pre-treatment hyponatremia has been associated with higher mortality in localized and metastatic renal cell carcinoma [22], malignant pleural mesothelioma [23], and small-cell lung cancer [24]. While findings reported here do not support this hypothesis, they do illustrate potential pitfalls with studies of serum levels of Na/K and PCa outcomes.

Results from these studies should be carefully interpreted as there are several concerns related to timing of serum sample collection and relevant exposure thresholds. For instance, if Na/K status was measured post-diagnosis or post-treatment, possibility of reverse causality and modification by treatment exist, but we did not observe an association with rapidly fatal PCa. However, even though we grouped Na/K status into clinically relevant categories, we did not have data to examine seasonally-adjusted Na/K status or a potential dose effect as to verify the assumption of a linear relationship between serum Na/K and prognosis [25–28]. Interestingly, Na/K exposure at PCa tissue microenvironment may not be a surrogate of commonly measured serum levels [29]. The lack of an association in our study might also be due to limited information on additional serum electrolytes like Magnesium (Mg) or single observations over a long interval time – however stratification by interval time did not alter our observations. To our knowledge no other study has yet investigated the role of electrolytes in PCa progression. Hence, further research is required to disentangle their role— pre-clinical studies on PCa, observational studies with repeated measurements, testing of causal relationship through linear as well as polynomial models, adding additional electrolytes like Mg, investigating the seasonal/ dietary factors involved in regulation serum electrolytes, etc.

To date, this is the largest prospective study assessing pre-diagnostic serum electrolytes in relation to PCa-specific death. There was limited information for potential covariates such as renal and endocrine functions or additional serum electrolytes. However, all models were adjusted for CCI. It is a limitation of our study that we did not have repeated measurements of Na and K, as this could have provided more information on the natural history of PCa. Another limitation is that we did not account for PCa treatment when studying mortality, however by considering disease severity at time of diagnosis we have taken into account the best proxy possible.

## Conclusion

We did not find any marked association between commonly measured serum electrolytes prior to diagnosis and risk of death from PCa, cardiovascular disease, or all-cause mortality. Future studies should investigate temporality of these associations using a modelling approach based on repeated measurement of electrolytes to provide more insight into these possible links.

## Abbreviations

AMORIS: Apolipoprotein-related MOrtality RISk
CALAB: Central Automation Laboratory
CCI: Charlson Comorbidity Index
CI: Confidence interval
HR: Hazard ratio
K: potassium
mEq/L: milliequivalents per litre
Mg: Magnesium
Na: sodium
NHANES: National Health and Nutrition Examination survey
PCa: prostate cancer
RCS: restricted cubic splines
SAS: Statistical Analysis System
SD: Standard deviation
μmol/L: micromole per litre

## Footnotes

1 risk categories were defined in accordance with an adapted version of National Comprehensive Cancer Network as low risk (T1–2, Gleason score 2–6 and PSA < 10 ng/mL), intermediate risk (T1–2, Gleason score 7 and/or PSA 10 to < 20 ng/mL), high risk (T3 and/or Gleason score 8–10 and/or PSA 20 to < 50 ng/mL), and regionally metastatic tumors (T4 and/or N1 and/or PSA 50–< 100 ng/mL in the absence of distant metastases (M0 or MX)) and distant metastatic tumors (M1 and/or PSA > 100 ng/mL) [12]
